# Real-World Outcomes of PET-Adapted Treatment for Classic Hodgkin’s Lymphoma: A Study From a Single Tertiary Care Center

**DOI:** 10.7759/cureus.83836

**Published:** 2025-05-10

**Authors:** Imran Iftikhar, Mansoor Abbas, Maryam Afzal, Hasan Bilal, Muhammad Uzair, Muhammad Qasim, Bushra Ahsan, Usman Ahmad, Syed Waqas Imam Bokhari

**Affiliations:** 1 Clinical Hematology, Shaukat Khanum Memorial Cancer Hospital and Research Centre, Lahore, PAK; 2 Medical Oncology, Shaukat Khanum Memorial Cancer Hospital and Research Centre, Lahore, PAK

**Keywords:** abvd, classic hodgkin's lymphoma, escalated beacopp, pet-adapted approach, real-world outcomes

## Abstract

Background

Classic Hodgkin’s lymphoma (CHL) is frequently treated using a positron emission tomography (PET)-directed approach, as demonstrated in the Response-Adapted Therapy for Hodgkin Lymphoma (RATHL) trial. This study aimed to compare real-world patient outcomes with those reported in the RATHL trial for individuals with advanced-stage disease.

Methods

This retrospective study included 169 adult patients aged 18 years and older (range 20-66 years). Only patients who received treatment and subsequent follow-up at our institution were included in this study.

Results

The study population had a male-to-female ratio of 1.86:1, with a median age of 30 years. B symptoms were present in 119 patients (70.4%), while bulky disease (>33% of the transthoracic diameter or >10 cm elsewhere) was observed in 55 patients (32.5%). More than half (57.4%) had stage IV disease at diagnosis, and the median follow-up time was 4.79 years. The three-year overall survival rate was 92.3%, and the progression-free survival rate was 76.9%. Among the 49 patients in the interim PET-positive group, the three-year overall survival and progression-free survival rates were 83.7% and 57.1%, respectively. Of these, 17 (34.7%) received escalated therapy with bleomycin, etoposide, doxorubicin, cyclophosphamide, vincristine, procarbazine, and prednisone (BEACOPP), while 32 (65.7%) continued with doxorubicin, bleomycin, vinblastine, and dacarbazine (ABVD) or standard BEACOPP. No significant survival difference was observed between these treatment groups. In the interim PET-negative group of 104 patients, 84 (80.8%) received ABVD, while 20 (19.2%) received doxorubicin, vinblastine, and dacarbazine (AVD). Again, no significant difference in survival was noted between these two groups. When comparing the interim PET-positive cohort to the RATHL trial, the three-year overall survival rates were 83.7% versus 87.8% (p = 0.45), and the progression-free survival rates were 57.1% versus 67.5% (p = 0.17), with no statistically significant difference.

Conclusion

This study highlights excellent real-world outcomes for treating CHL using a PET-directed approach similar to the RATHL trial. However, despite PET-guided therapy, interim PET positivity remained associated with significantly lower overall survival and progression-free survival rates. De-escalation to AVD in the interim PET-negative group did not negatively affect survival outcomes. ABVD remains a viable treatment option for PET-positive patients with good tolerance, strong response, or near-complete remission with single-site residual disease, without compromising survival.

## Introduction

Classic Hodgkin's lymphoma (CHL) is the most common type of lymphoma, predominantly affecting middle-aged individuals and often interrupting their most productive years [1]. Despite its impact, CHL remains one of the most curable malignancies, demonstrating a high response rate to chemotherapy, chemoimmunotherapy, and radiotherapy [2].

The diagnosis of CHL is established through a lymph node biopsy, which allows for the morphological classification into four subtypes: nodular sclerosis, lymphocyte-rich, lymphocyte-depleted, and mixed cellularity [3]. Immunohistochemical staining further aids in confirming the diagnosis. Staging is performed using the Ann Arbor staging system, categorizing early-stage patients (stages I and II) as favorable or unfavorable based on the European Organization for Research and Treatment of Cancer (EORTC)/German Hodgkin Study Group (GHSG) criteria. For advanced stages (III and IV), the Hasenclever International Prognostic Score (IPS) is utilized to assess prognosis [4,5].

Treatment regimens for CHL typically include combinations of Adriamycin, bleomycin, vinblastine, and dacarbazine (ABVD) or bleomycin, etoposide, doxorubicin, cyclophosphamide, vincristine, procarbazine, and prednisone (BEACOPP), administered at standard or escalated doses. Positron emission tomography-computed tomography (PET-CT) plays a crucial role in response assessment, guiding treatment modifications based on interim results. A PET-adapted approach has been shown to mitigate toxicity through de-escalation when appropriate and enhance response rates through escalation when necessary [5-7].

The Response-Adapted Therapy in Hodgkin Lymphoma (RATHL) trial (2008-2012) evaluated PET-adapted treatment strategies for CHL patients meeting specific eligibility criteria. Participants initially received two cycles of ABVD, followed by an interim PET scan. Patients with a Deauville score of 1-3 either continued ABVD or had bleomycin omitted (Adriamycin, vinblastine, dacarbazine (AVD)) to minimize toxicity. Overall survival and progression-free survival outcomes were subsequently compared between these groups. PET-positive patients (Deauville scores of 4-5) were escalated to either BEACOPP-14 or escalated BEACOPP, receiving three cycles of escalated BEACOPP (three-week cycles) and four of BEACOPP-14 (two-week cycles). A repeat PET scan was then performed. Those who became PET-negative and had received BEACOPP-14 were given two additional cycles per the RATHL protocol, while those treated with escalated BEACOPP received one more cycle. Patients with persistent positive scans were considered for salvage therapy. An end-of-treatment PET scan was conducted, and radiotherapy was administered to sites of bulky disease as indicated [8].

## Materials and methods

Study design and participants

This retrospective study aimed to describe patient demographics, the prevalence of advanced-stage disease (stage III, stage IV, stage II-B, and stage II-A with adverse factors), and to correlate these factors with treatment outcomes in patients treated with the RATHL approach. All consecutive eligible patients diagnosed with CHL and treated at Shaukat Khanum Memorial Cancer Hospital and Research Centre (SKMCH&RC) between January 1, 2019, and January 1, 2021, were included in the study. Patients with nodular lymphocyte-predominant Hodgkin's lymphoma (NLPHL) and early-stage CHL, those who did not undergo interim scans, and those receiving treatments other than the RATHL approach were excluded from the research. Initially, 230 patients diagnosed with CHL were identified. Since the study aimed to assess survival outcomes in patients treated with the RATHL approach, only 169 patients met the eligibility criteria. The final study population comprised both male and female patients aged 18 years or older with histologically confirmed CHL; specifically, an advanced stage was defined as an Ann Arbor stage of IIB to IV or stage IIA with adverse features: bulky disease (>33% of the transthoracic diameter or >10 cm elsewhere) or at least three involved sites. Patients with insufficient data were also excluded from the analysis. The study was approved as a retrospective study by the Institutional Review Board (IRB) of SKMCH&RC (EX-05-09-22-01), which also granted a waiver of informed consent. The data used in the analysis was anonymized.

Methods

Information regarding demographic distribution, baseline characteristics, clinical and pathological parameters, and PET-CT reports was obtained from patients’ medical records available in the electronic hospital information system. The interim PET response, whether partial or complete metabolic response, was confirmed using existing reports. PET scans with Deauville scores 1-3 were considered negative. Those with scores 4-5 were considered positive. The data collection cut-off date was December 5, 2024, at which point all surviving patients were censored. The main treatments included in the study were ABVD- and BEACOPP-escalated. Patients who received standard BEACOPP-14 due to the unavailability of dacarbazine were also included. The follow-up outcomes assessed were overall survival (OS) and progression-free survival (PFS). Follow-up data were collected from electronic patient records. Overall survival was determined based on the time interval between the date of diagnosis and the date of the last follow-up, with patient status at the last follow-up recorded as alive, deceased, or unknown. PFS was defined as the time from the start of treatment until disease progression, relapse, or death from any cause, whichever occurred first.

Statistical analysis

Data were entered into IBM SPSS Statistics for Windows, version 27.0 (released 2020, IBM Corp., Armonk, NY) for analysis. Major toxicities experienced by patients were analyzed and tabulated. Responses to interim scans, post-chemotherapy scans, and post-radiotherapy scans were also assessed. OS and PFS were calculated for the entire cohort. Given the median follow-up time of 4.79 years, three-year and five-year survival rates were determined. Survival outcomes were compared based on International Prognostic Score (IPS) categories. Univariate analysis was conducted using Kaplan-Meier methodology for categorical variables. Statistical significance was assessed via the log-rank test. Univariate analysis for continuous variables was done using Cox regression. Multivariate analysis was performed using Cox regression to adjust for confounding variables. Throughout the study, a p-value of less than 0.05 was considered statistically significant.

A second phase of the analysis focused on patients who underwent an interim PET scan, while those who had CT-NCAP imaging were excluded. Patients with Deauville scores of 1-3 were classified as PET-negative, whereas those with scores of 4-5 were considered PET-positive. Survival times were compared based on interim PET response status (positive vs. negative), following the same statistical methods applied to the entire cohort.

At our center, patients with a positive interim PET scan were evaluated for treatment escalation to escalated BEACOPP. However, those with tolerance issues or a near-complete metabolic response with only a small residual single-site lesion continued on ABVD. All other PET-positive patients were escalated to BEACOPP-escalated. Survival outcomes were compared between the escalated and non-escalated groups.

Finally, real-world data from our study were compared with the results of the RATHL trial, and conclusions were drawn accordingly.

## Results

A total of 169 patients met the inclusion criteria. The percentage of males was higher than that of females, with a male-to-female ratio of 1.86:1. B symptoms were observed in 70.4% of the patients at presentation. A total of 114 patients (67.5%) did not have bulky disease. More than half of the cohort (57.4%) had stage IV disease. The most common histological type was mixed cellularity, found in 48.5% of patients, followed by nodular sclerosis, which was observed in 34.3% of patients. The International Prognostic Score (IPS) was calculated for 135 patients (79.9%) with stage III and stage IV disease. IPS was not calculated for the 34 patients with stage II disease. Patient demographics and disease characteristics are summarized in Table 1.

**Table 1:** Demographics and disease characteristics. *IPS: International Prognostic Score

Variables	Categories	Frequency (n)	Percentage (%)
Gender	Males	110	65.1%
	Females	59	34.9%
B symptoms	Present	119	70.4%
	Absent	50	29.6%
Disease bulk	Bulky disease	55	32.5%
	Non-bulky disease	114	67.5%
Ann Arbor stage	Stage IIA	10	5.9%
	Stage IIB	24	14.2%
	Stage III	38	22.5%
	Stage IV	97	57.4%
Histological subtype	Mixed cellularity	82	48.5%
	Nodular sclerosis	58	34.3%
	Lymphocyte rich	3	1.8%
	Type not specified	26	15.4%
IPS^*^ (n = 135)	0-1	23	17%
	2	30	22.2%
	3	30	22.2%
	4	28	20.7%
	> 5	24	17.8%

Treatment was initiated with a median delay of 25 days (interquartile range: 17-38 days). A total of 125 patients (74%) received six cycles of ABVD. Eleven patients (6.5%) received two cycles of BEACOPP before an interim scan (due to the unavailability of Dacarbazine), followed by four cycles of ABVD. The number of ABVD and BEACOPP cycles administered varied depending on drug availability. Seventeen patients (10.1%) were escalated to BEACOPP. The details of the chemotherapy regimens administered to the patients are provided in Table 2.

**Table 2:** Frequencies and percentages of various chemotherapy cycles. *ABVD: Adriamycin (doxorubicin), bleomycin, vinblastine, dacarbazine. **BEACOPP: bleomycin, etoposide, Adriamycin (doxorubicin), cyclophosphamide, Oncovin (vincristine), procarbazine, prednisone. ***BEACOPP-escalated: escalated doses of bleomycin, etoposide, Adriamycin (doxorubicin), cyclophosphamide, Oncovin (vincristine), procarbazine, prednisone.  ~Interim PET scan: interim positron emission tomography scan.

Treatment	Frequency (n)	Percentage (%)
ABVD* x 6	125	74 %
BEACOPP** x 2 ; ABVD x 4	11	6.5%
ABVD x 2 ; BEACOPP-escalated*** x 4	16	9.5%
BEACOPP x 2 ; BEACOPP-escalated x 4	1	0.6%
ABVD x 5 ; BEACOPP x 1	2	1.2%
ABVD x 4 ; BEACOPP x 2	6	3.5%
ABVD x 3 ; BEACOPP x 3	5	2.9%
ABVD x 2 ; BEACOPP x 4	1	0.6%
ABVD x 1 ; BEACOPP x 5	2	1.2%
Patients escalated after interim PET scan~	17	10.1%

Response rates

After two cycles of treatment, 16 patients (9.5%) underwent CT-NCAP, while 153 patients (90.5%) underwent interim PET scans. In interim assessments (combined CT-NCAP and PET), 113 patients (66.9%) achieved a complete response (CR), 55 patients (32.5%) showed a partial response (PR), and progressive disease was noted in one patient (0.6%). The response rates of various treatment regimens are detailed in Table 3.

**Table 3:** Response rates on interim scans. *ABVD: Adriamycin (doxorubicin), bleomycin, vinblastine, dacarbazine. **BEACOPP: bleomycin, etoposide, Adriamycin (doxorubicin), cyclophosphamide, Oncovin (vincristine), procarbazine, prednisone. ***BEACOPP-escalated: escalated doses of bleomycin, etoposide, Adriamycin (doxorubicin), cyclophosphamide, Oncovin (vincristine), procarbazine, prednisone.

Treatments offered	Complete response (CR)	Partial response (PR)	Disease progression
ABVD* x 6 N = 125	97 (77.6%)	28 (22.4%)	0
BEACOPP **x 2 ; ABVD x 4 N = 11	10 (90.9%)	1 (9.1%)	0
ABVD x 2 ; BEACOPP-escalated*** (n = 16)	0	16 (100%)	0
BEACOPP x 2 ; BEACOPP-escalated (n = 1)	0	1 (100%)	0
ABVD x 5 ; BEACOPP x 1 (n = 2)	2 (100%)	0	0
ABVD x 4 ; BEACOPP x 2 (n = 6)	3 (50%)	2 (33.3%)	1 (16.7%)
ABVD x 3 ; BEACOPP x 3 (n = 5)	1 (20%)	4 (80%)	0
ABVD x 2 ; BEACOPP x 4 (n = 1)	0	1 (100%)	0
ABVD x 1 ; BEACOPP x 5 (n = 2)	0	2 (100%)	0
Overall responses	113 (66.9%)	55 (32.5%)	1 (0.6%)

A total of 104 patients (61.5%) had end-of-chemotherapy PET scans. Thirty-one patients (18.3%) underwent post-radiotherapy PET scans, and 24 patients (14.2%) had both post-chemotherapy and post-radiotherapy PET scans. Ten patients (5.9%) had no scans after chemotherapy or radiotherapy. Complete metabolic response (CMR) was observed in 96 patients (75%), partial metabolic response (PMR) in 14 patients (10.9%), and disease progression in 18 patients (14.1%). End-of-treatment responses are summarized in Table 4.

**Table 4:** Response rates on end-of-chemotherapy PET scans. *ABVD: Adriamycin (doxorubicin), bleomycin, vinblastine, dacarbazine. **BEACOPP: bleomycin, etoposide, Adriamycin (doxorubicin), cyclophosphamide, Oncovin (vincristine), procarbazine, prednisone. ***BEACOPP-escalated: escalated doses of bleomycin, etoposide, Adriamycin (doxorubicin), cyclophosphamide, Oncovin (vincristine), procarbazine, prednisone.

Treatment	Complete metabolic response (CMR)	Partial metabolic response (PMR)	Disease progression
ABVD* x 6 (n = 95)	73 (76.8%)	9 (9.5%)	13 (13.7%)
BEACOPP** x 2 ; ABVD x 4 (n = 8)	6 (75%)	1 (12.5%)	1 (12.5%)
ABVD x 2 ; BEACOPP-escalated*** (n = 11)	8 (72.7%)	1 (9.1%)	2 (18.2%)
BEACOPP x 2 ; BEACOPP-escalated (n = 1)	0	1 (100%)	0
ABVD x 5 ; BEACOPP x 1 (n = 2)	2 (100%)	0	0
ABVD x 4 ; BEACOPP x 2 (n = 4)	3 (75%)	1 (25%)	0
ABVD x 3 ; BEACOPP x 3 (n = 5)	3 (60%)	1 (20%)	1 (20%)
ABVD x 2 ; BEACOPP x 4 (n = 1)	1 (100%)	0	0
ABVD x 1 ; BEACOPP x 5 (n = 1)	0	0	1 (100%)
Overall responses	96/128 (75%)	14/128 (10.9%)	18/128 (14.1%)

A total of 65 patients (38.5%) received radiotherapy, while 104 (61.5%) did not. Fifty-five patients (84.6%) received radiotherapy due to bulky disease, and 10 patients (15.4%) due to residual disease. Of the 65 patients who received radiotherapy, 55 (84.6%) underwent post-radiotherapy PET scans, which represented 32.5% of the total cohort. Among these 55 patients, CMR was achieved in 43 (78.2%), PMR in five (9.1%), and disease progression in seven (12.7%) patients. Post-radiotherapy PET responses are detailed in Table 5.

**Table 5:** Response rates on post-radiotherapy PET scans. *ABVD: Adriamycin (doxorubicin), bleomycin, vinblastine, dacarbazine. **BEACOPP: bleomycin, etoposide, Adriamycin (doxorubicin), cyclophosphamide, Oncovin (vincristine), procarbazine, prednisone. ***BEACOPP-escalated: escalated doses of bleomycin, etoposide, Adriamycin (doxorubicin), cyclophosphamide, Oncovin (vincristine), procarbazine, prednisone.

Treatment	Complete metabolic response (CMR)	Partial metabolic response (PMR)	Disease progression
ABVD* x 6 (n = 40)	35 (87.5%)	1 (2.5%)	4 (10%)
BEACOPP **x 2 ; ABVD x 4 (n = 5)	4 (80%)	1 (20%)	0
ABVD x 2 ; escBEACOPP*** (n = 7)	3 (42.8%)	2 (28.6%)	2 (28.6%)
BEACOPP x 2 ; BEACOPP-escalated (n = 1)	0	1 (100%)	0
ABVD x 4 ; BEACOPP x 2 (n = 2)	1 (50%)	0	1 (50%)
Total	43/55 (78.2%)	5/55 (9.1%)	7/55 (12.7%)

**Figure 1:**
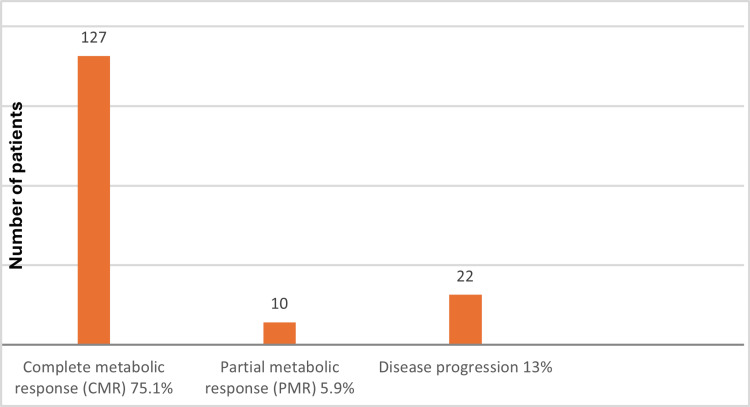
Overall response rates (ORR) to first-line therapy.

Overall, 127 patients (75.1%) achieved CMR following first-line chemotherapy. PMR was observed in 10 patients (5.9%), and disease progression was observed in 22 patients (13%). Approximately three-fourths of the patients achieved CMR, followed by disease progression in less than one-fourth of the population. PMR was observed in the fewest number of patients. Response rates are illustrated in Figure 1.

Survival outcomes

Data were collected for patients diagnosed from January 3, 2019, to January 11, 2021. The data analysis cut-off date was December 5, 2024. The median follow-up duration for the cohort was 4.79 years (interquartile range = 4.22-5.46 years). During the follow-up period, there were 15 deaths (8.9%), while 154 patients (91.1%) were alive at their last follow-up. Six deaths were attributable to the disease. Details on the causes of death are provided in Table 6.

**Table 6:** Causes of death.

Cause of death	Frequency (n)	Percentage (%)
Disease related	6	40%
Infections	7	46.6%
Bilateral pulmonary embolisms	1	6.7%
Unknown	1	6.7%

**Figure 2:**
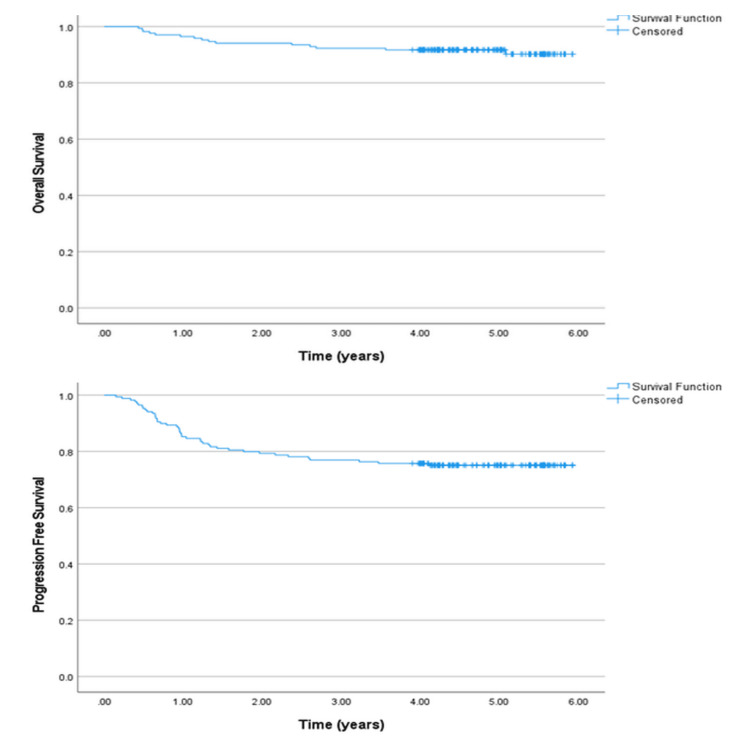
Overall survival (OS) and progression-free survival (PFS) for the whole study cohort.

OS and PFS were calculated for the entire cohort. Three-year OS and five-year OS were 92.3% and 91.7%, respectively. Three-year PFS and five-year PFS were 76.9% and 75.1%, respectively. The Kaplan-Meier curves for OS and PFS are shown in Figure 2.

Univariate and multivariate analyses

Univariate and multivariate analyses were performed to assess variables affecting survival outcomes (OS and PFS) for the entire cohort. In the univariate analysis of the whole cohort, the Ann Arbor stage (p = 0.03) and interim PET response (p = 0.01) significantly impacted OS. Treatment escalation (p = 0.01) and interim PET response (p < 0.001) significantly affected PFS. In the multivariate analysis of the whole cohort, radiotherapy (p = 0.02) and bulky disease (p = 0.003) significantly affected OS. However, none of the factors significantly impacted PFS. No statistically significant impact of the International Prognostic Score (IPS) on OS or PFS was observed, with p-values of 0.38 for OS and 0.23 for PFS. The three- and five-year OS and PFS for IPS are compared in Table 7.

**Table 7:** Comparison of overall survival (OS) and progression-free survival (PFS) for the International Prognostic Score (IPS).

International Prognostic Score (IPS)	Overall survival (OS)		Progression-free survival (PFS)	
	Three-year OS	Five-year OS	Three-year PFS	Five-year PFS
0-1	91.3%	91.3%	82.6%	78.3%
2	96.7%	96.7%	86.7%	86.7%
3	83.3%	83.3%	63.3%	60%
4	100%	96.4%	75%	75%
>5	83.3%	83.3%	70.8%	66.4%

**Figure 3:**
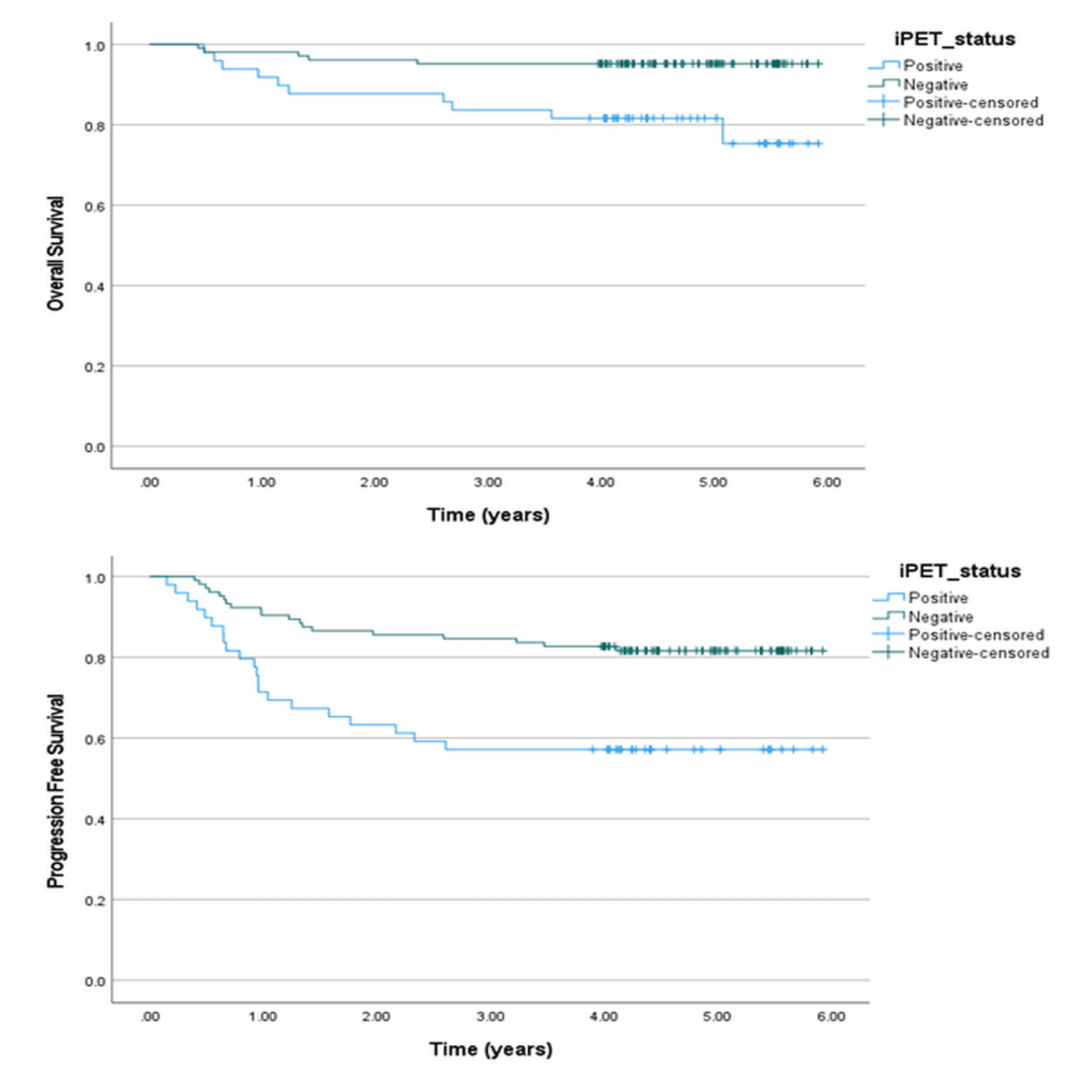
Overall survival (OS) and progression-free survival (PFS) curves compared for the interim PET status. iPET: interim positron emission tomography scan

Next, univariate and multivariate analyses were also conducted for the subgroup of patients who underwent interim PET scans, excluding those who had CT-NCAP. In the univariate analysis, the Ann Arbor stage (p = 0.02) and interim PET response (p = 0.002) significantly affected OS. Treatment escalation (p = 0.03), delays in treatment (p = 0.001), and interim PET response (p < 0.001) significantly affected PFS. For the cohort with positive interim PET scans, three-year and five-year OS were 83.7% and 81.6%, respectively. For those who had negative interim PET scans, the three-year and five-year OS rates were 95.2%. For the patients with positive interim PET scans, three-year and five-year PFS were 57.1%. For those who had negative interim PET scans, three-year and five-year PFS were 84.6% and 81.6%, respectively. The Kaplan-Meier curves for OS and PFS are shown in Figure 3.

In the multivariate analysis of the subgroup of patients who had interim PET scans, bulky disease (p = 0.006), interim PET result (p = 0.007), and radiotherapy (p = 0.02) significantly affected OS, while interim PET results (p-value = 0.005) significantly affected PFS. The Ann Arbor stage, treatment escalation, and delays in treatment did not show statistically significant associations with OS and PFS in multivariate analysis. The results of the univariate and multivariate analyses are shown in Table 8.

**Table 8:** Summary of the univariate and multivariate analyses for the whole study cohort and subgroup of patients who underwent interim PET scans. *IPS: International Prognostic Score, PET: positron emission tomography

Variables	Univariate analysis		Multivariate analysis	
	Overall survival (p-value)	Progression-free survival (p-value)	Overall survival (p-value)	Progression-free survival (p-value)
Analysis for the whole study cohort				
Radiotherapy	0.31	0.72	0.02	0.09
Age	0.92	0.09	0.34	0.29
Gender	0.94	0.89	0.86	0.91
B symptoms	0.41	0.96	0.43	0.63
Bulky disease	0.06	0.20	0.003	0.08
Ann Arbor stage	0.03	0.40	0.42	0.23
Histological subtype	0.57	0.05	0.51	0.70
IPS^*^	0.38	0.23	0.13	0.24
Treatment offered – escalation vs. no escalation	0.13	0.01	0.71	0.27
Interim scan responses	0.01	<0.001	0.07	0.11
Dose reduction (doxorubicin)	0.79	0.98	0.52	0.62
Dose reduction (bleomycin)	0.95	0.17	0.48	0.05
Dose reduction (vinblastine)	0.69	0.24	0.22	0.18
Dose reduction (dacarbazine)	0.75	0.38	0.84	0.12
Treatment delays	0.78	0.82	0.74	0.96
Analysis for the interim PET cohort				
Age	0.08	0.08	0.47	0.16
Gender	0.90	0.59	0.88	0.50
B symptoms	0.45	0.93	0.66	0.51
Ann Arbor stage	0.02	0.45	0.96	0.50
Histological subtype	0.68	0.09	0.99	0.09
Bulky disease	0.06	0.11	0.006	0.06
IPS^*^	0.35	0.50	0.11	0.08
interim PET status	0.002	<0.001	0.007	0.005
Treatment offered: escalated vs. not escalated	0.19	0.03	0.94	0.50
Radiotherapy	0.27	0.53	0.02	0.16
Dose reduction (doxorubicin)	0.79	0.95	0.55	0.85
Dose reduction (bleomycin)	0.89	0.25	0.40	0.14
Dose reduction (vinblastine)	0.59	0.26	0.25	0.21
Dose reduction (dacarbazine)	0.80	0.60	0.73	0.13
Treatment delays	0.80	0.001	0.96	0.92

Next, we carried out an analysis on the patients who had positive interim PET scans. A total of 49 patients had positive interim PET scans. Seventeen patients (34.7%) were escalated to BEACOPP-escalated. Twenty-two out of 32 patients did not receive BEACCOPP-escalated because they had a single-site disease. The summary is shown in Table 9.

**Table 9:** Proportion of patients receiving BEACOPP-escalated and reasons for continuation of ABVD. *Escalated: BEACOPP-escalated, escalated doses of bleomycin, etoposide, Adriamycin, cyclophosphamide, vincristine, procarbazine, and prednisone. ** Not escalated: These patients were either continued on ABVD (Adriamycin (doxorubicin), bleomycin, vinblastine, and dacarbazine) or baseline BEACOPP (bleomycin, etoposide, Adriamycin, cyclophosphamide, vincristine, procarbazine, and prednisone).

Variables	Category	Frequency (n)	Percentage (%)
Escalation status	Escalated to BEACOPP-escalated^*^	17	34.7%
	Not escalated^**^	32	65.3%
Reason for not escalating	Single-site disease	22	68.8%
	Tolerance issues	10	31.2%

**Figure 4:**
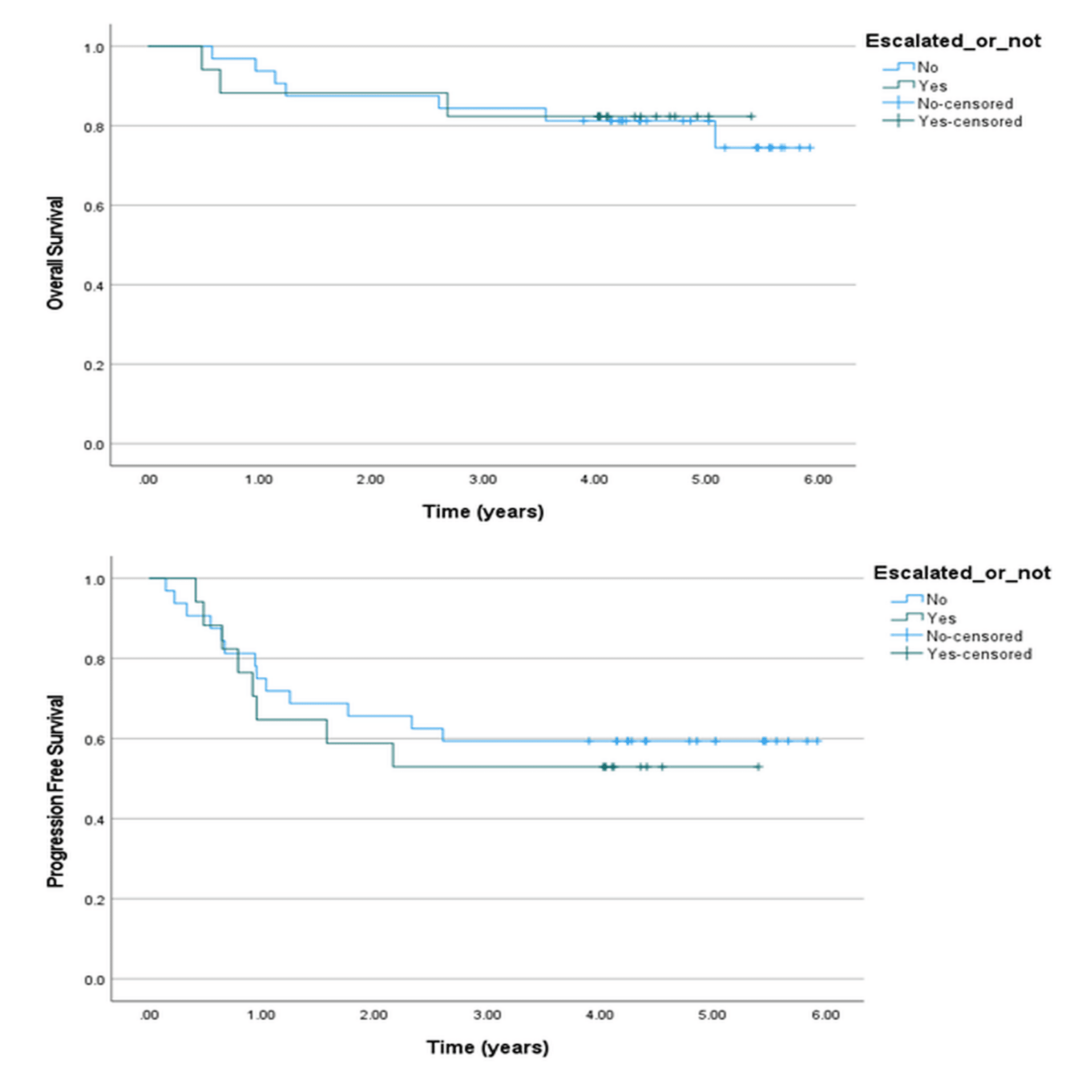
Overall survival (OS) and progression-free survival (PFS) curves compared for treatment escalation.

We divided the patients with positive interim PET scans into two groups and compared OS and PFS between those who received BEACOPP-escalated treatment and those who continued with ABVD or baseline chemotherapy. No statistically significant differences were found. Patients who received BEACOPP-escalated had three-year and five-year OS of 82.4%. By contrast, patients who were not escalated had three-year and five-year OS of 84.4% and 81.3%, respectively. The p-value for OS was 0.92. The three-year and five-year PFS for patients who received BEACOPP-escalated was 52.9%, while those who were not escalated had three-year and five-year PFS of 59.4%. The p-value for PFS was 0.66. Kaplan-Meier curves for OS and PFS are shown in Figure 4.

We compared the demographics and disease characteristics between the patient groups with positive interim PET scans who could and could not be escalated. For continuous variables such as age, we compared medians. Chi-square test and Fisher’s exact test were used for categorical variables, as appropriate. In the group treated with BEACOPP-escalated, the median age was 29 years, which was not significantly different from the group that continued on baseline chemotherapy (27 years). Most of the patients who received escalation were male (13 out of 17; 76.5%). Twenty patients out of 32 who were not escalated were male (62.5%). Gender did not have a statistically significant impact on the treatment decision regarding escalation. Both groups had a relatively higher percentage of patients with B symptoms. This percentage was 88.2% in the group treated with BEACOPP-escalated and 75% in the other group. This difference had no statistical significance. Bulky disease, IPS, and histological subtype did not have any statistically significant impact on the decision to escalate as well. We did find a significant association between the Ann Arbor stage and escalation. We observed that 88.2% of the patients in the BEACOPP-escalated group had a stage IV disease, compared to 62.5% in the non-escalated group. The p-value for this association was 0.02.

**Figure 5:**
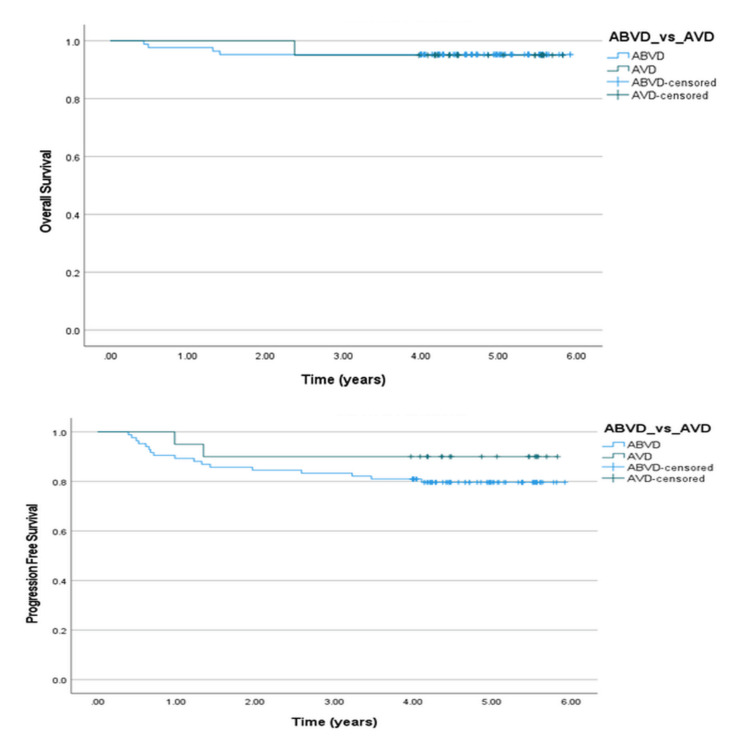
Overall survival (OS) and progression-free survival (PFS) curves compared for ABVD vs. AVD. ABVD: Adriamycin (doxorubicin), bleomycin, vinblastine, dacarbazine. AVD: Adriamycin (doxorubicin), vinblastine, and dacarbazine.

Next, we compared survival proportions for patients with negative interim PET scans who were managed either by omission of bleomycin (AVD) or by continuation of ABVD. There was no significant effect on OS or PFS. The three-year and five-year OS for patients who received either ABVD or AVD chemotherapy was 95.2% and 95%, respectively. The p-value for OS was 0.98. Three-year and five-year PFS of the patients who continued ABVD chemotherapy were 83.3% and 79.6%, respectively. The three-year and five-year PFS of the patients who had de-escalation to AVD was 90%. The p-value for PFS was 0.29. The Kaplan-Meier curves for OS and PFS are shown in Figure 5.

For the interim PET negative cohort, none of the factors significantly affected OS. PFS was only affected by IPS (p = 0.02). Multivariate analysis showed no effect of any variable on OS. PFS was affected by bulky disease (p = 0.01), radiotherapy (p = 0.01), and dose reduction in dacarbazine (p = 0.04).

Infections were observed in 37 patients (21.9%). Twenty-nine patients had pulmonary side effects. The next most common side effects were neutropenic colitis (including one case of *Clostridium difficile* colitis) and mucositis, seen in 22 patients each. Fifteen patients had gastrointestinal (GI) side effects. Vomiting was observed in eight patients, and hematemesis in one patient. Four patients had gastritis. Constipation, rectal bleeding, and xerostomia were observed in one patient each. Among the patients who had cytopenias, 14 patients had neutropenia and three had anemia. Only two patients (1.2%) had central nervous system side effects, including insomnia and convulsions. Major toxicities observed in the study population are summarized in Table 10. 

**Table 10:** Toxicities observed in the study population

Toxicities	Frequency (n)	Percentage (%)
Infections	37	21.9%
Hematologic toxicities	17	10.1%
Febrile neutropenia	13	7.7%
Neutropenic sepsis	6	3.6%
Neutropenic colitis	22	13%
Hepatotoxicity	10	5.9%
Pulmonary toxicity	29	17.2%
Cardiotoxicity	4	2.4%
Peripheral neuropathy	8	4.7%
Central nervous system toxicity	2	1.2%
Mucositis	22	13%
Electrolyte imbalance	4	2.4%
Acute kidney injury	1	0.6%
Vascular side effects	4	2.4%
Myalgias	2	1.2%
Dacarbazine infusion reactions	4	2.4%
Gastrointestinal toxicity	15	8.9%

Skin infections were observed in 10 out of 37 patients (27.1%), including two cases (5.4%) of fungal skin infections. Six patients (16.2%) developed lower respiratory tract infections; among them, one had a *Klebsiella pneumoniae* infection, and one was diagnosed with pulmonary tuberculosis. Urinary tract infections (UTIs) occurred in five patients (13.5%), with *Escherichia coli* isolated in the urine of one patient. The frequencies and percentages of the various types of infections observed in the study population are shown in Table 11. 

**Table 11:** Types of infections observed in the study population. *NA: not applicable

Types of infection	Total n (%)	Subgroups	Subgroups frequency (n) and precentage (%)
Urinary tract infections	5 (13.5%)	*Escherichia coli* infection	1/5 (20%)
Upper respiratory tract infections	1 (2.7%)	None	N/A^*^
Lower respiratory tract infections	6 (16.2%)	*Klebsiella pneumoniae* infection	1/6 (16.7%)
		Tuberculosis	1/6 (16.7%)
		Atypical pneumonia	1/6 (16.7%)
COVID	4 (10.8%)	None	N/A^*^
Skin infections	10 (27.1%)	Fungal skin infections	2/10 (20%)
Shingles	2 (5.4%)	None	N/A^*^
Oral thrush	3 (8.1%)	None	N/A^*^
Abscess	3 (8.1%)	None	N/A^*^
Pharyngitis / tonsillitis	1 (2.7%)	None	N/A^*^
Submandibular gland infection	1 (2.7%)	None	N/A^*^
Necrotizing fascitis	1 (2.7%)	None	N/A^*^

Comparison with the RATHL trial

The demographics, disease characteristics, and treatment data are compared for our study and the RATHL trial. The total number of patients who met the inclusion criteria and were eligible for inclusion in our study and the RATHL trial was 169 and 1203, respectively. The male-to-female ratio in our study group was 1.86:1, whereas in the RATHL trial, this ratio was 1.2:1. The p-value for this difference was 0.009. In our study population, stage II, stage III, and stage IV diseases were observed in 20.1%, 22.5%, and 57.4% of patients, respectively. In the RATHL trial, the percentages of stage II, stage III, and stage IV patients were 41.6%, 30.2%, and 28.3%, respectively. B symptoms were more common in our study population (70.4%) as compared to the RATHL trial (61.3%). A total of 937 patients (83.7%) achieved CMR on interim PET scans in the RATHL compared to 66.9% of patients in our study. A total of 465 patients (49.6%) received AVD in the RATHL trial. In our study, the percentage of these patients was only 19.2%. In the interim PET-positive group, only 17 patients (34.7%) were escalated to BEACOPP-escalated. This percentage was significantly lower than that of the RATHL trial. The summary is tabulated in Table 12.

**Table 12:** Raw data comparison between our study and the RATHL trial. *N/A: not applicable. **CR: complete response. ***PR: partial response. ****ABVD: Adriamycin (doxorubicin), bleomycin, vinblastine, dacarbazine. *****AVD: Adriamycin (doxorubicin), vinblastine, dacarbazine .~BEACOPP: bleomycin, etoposide, adriamycin, cyclophosphamide, Oncovin (vincristine), procarbazine, prednisone. ~~BEACOPP-escalated: escalated-dose bleomycin, etoposide, adriamycin, cyclophosphamide, Oncovin (vincristine), procarbazine, prednisone. ~~~BEACOPP-14: 14-day cycle of bleomycin, etoposide, adriamycin, cyclophosphamide, Oncovin (vincristine), procarbazine, prednisone. RATHL: Response-Adapted Therapy for Hodgkin's Lymphoma

Parameters	Categories	Our study	RATHL Trial	z-score	p-value
Total patients	N/A*	169	1214	-	-
Eligible patients	N/A*	169	1203	-	-
Median age (years)	N/A*	30	33	-	-
Gender	Males	110 (65.1%)	656 (54.5%)	2.60	0.009
	Females	59 (34.9%)	547 (45.5%)		
Ann Arbor stage	Stage II	34 (20.1%)	500 (41.6%)	-5.37	0.000
	Stage III	38 (22.5%)	363 (30.2%)	-2.06	0.04
	Stage IV	97 (57.4%)	340 (28.3%)	7.61	<0.00001
B symptoms	N/A*	119 (70.4%)	738 (61.3%)	2.29	0.022
Bulky disease	N/A*	55 (32.5%)	386 (32.1%)	0.10	0.917
International Prognostic Score (IPS)	0-1	23 (13.5%)	404 (33.6%)	-5.28	0.000
	2 or 3	60 (35.4%)	579 (48.1%)	-3.10	0.001
	>4	52 (30.7%)	209 (17.4%)	4.12	0.000
	Missing	34 (20.4%)	11 (0.9%)	7.60	-
Interim scan results	Total interim scans	169	1119	-	-
	CR** (Deauville score 1-3)	113 (66.9%)	937 (83.7%)	-5.24	0.000
	PR*** (Deauville score 4)	55 (32.5%)	144 (12.9%)	6.57	0.000
	Disease progression (Deauville score 5)	1 (0.6%)	38 (3.4%)	-1.98	0.048
Treatment groups of interim PET-negative patients	Total	104	937	-	-
	Not treated	0	2 (0.2%)	-	-
	ABVD****	84 (80.8%)	470 (50.2%)	2.97	0.000
	AVD*****	20 (19.2%)	465 (49.6%)	-5.90	0.000
	Radiotherapy	36 (34.6%)	32 (3.4%)	12.22	0.000
Treatment groups of interim PET-positive patients	Total	49	182	-	-
	Not treated	0	6 (3.3%)	-	-
	ABVD	21 (42.9%)	4 (2.2%)	8.05	0.000
	ABVD + BEACOPP~	11 (22.4%)	0	-	-
	BEACOPP-escalated~~	17 (34.7%)	78 (42.9%)	-9.37	0.000
	BEACOPP-14~~~	0	94 (51.6%)		
	Radiotherapy	24 (49%)	20 (10.9%)	6.04	0.000

A comparison of survival proportions was done with the RATHL trial. Three-year OS and three-year PFS were compared. The p-value for OS of the whole cohort was significant (0.04), indicating that the OS of our study population was shorter than that in the RATHL trial (92.3% vs. 95.8%). The comparison of three-year PFS did not show a significant p-value (0.07). OS and PFS comparisons for the group that received ABVD chemotherapy did not show a statistically significant difference. The p-values for OS and PFS were 0.33 and 0.56, respectively. The comparison of OS and PFS for the AVD group showed p-values of 0.47 and 0.50, respectively. The OS and PFS of patients in the group with positive interim PET results compared to the RATHL trial did not show a statistically significant difference. The p-values for OS and PFS were 0.45 and 0.17, respectively. The comparison of survival proportions with the RATHL trial is presented in Table 13.

**Table 13:** Comparison of survival proportions of our study with the RATHL trial. *OS: overall survival. **PFS: progression-free survival. ^***^Interim positron emission tomography. ^~^ABVD: Adriamycin (doxorubicin), bleomycin, vinblastine, dacarbazine. ^~~^AVD: Adriamycin (doxorubicin), vinblastine, dacarbazine. RATHL: Response-Adapted Therapy for Hodgkin's Lymphoma

Study population	Survival functions	Our study	RATHL trial	Z-score	P-value
Whole cohort	Three-year OS*	92.3%	95.8%	-2.03	0.04
	Three-year PFS**	76.9%	82.6%	-1.80	0.07
ABVD^~^ group	Three-year OS	95.2%	97.2%	-0.97	0.33
	Three-year PFS	83.3%	85.7%	-0.57	0.56
AVD^~~^ group	Three-year OS	95%	97.6%	-0.73	0.47
	Three-year PFS	90%	84.4%	0.67	0.50
Interim PET^***^-positive group	Three-year OS	83.7%	87.8%	-0.75	0.45
	Three-year PFS	57.1%	67.5%	-1.36	0.17

## Discussion

We used a PET-adapted approach in treating CHL. Several studies have highlighted the significance of PET-guided therapy, notably Mahuad C et al. [9]. The median age in our patient cohort was 30 years, consistent with the findings reported by Borchmann P et al. [10]. The male-to-female ratio was 1.86:1, reflecting a predominance of male patients - a trend similarly observed by Bhurani D et al.in India, although their study noted a higher median age [11]. The most prevalent histological subtype in our group was mixed cellularity, which was followed by lymphocyte-rich CHL and nodular sclerosis. A study from Pakistan reported a similar distribution of subtypes [12]. About 66% of our patients had non-bulky illness, and more than 66% had B symptoms. Most of our patients were diagnosed at stage IV.

Seventy-five percent of patients received ABVD as the first-line therapy. However, due to the unavailability of dacarbazine, approximately 7% were treated with BEACOPP. These variations in the initial treatment did not result in statistically significant differences in outcomes. The three-year and five-year OS rates were 92.3% and 91.7%, respectively. The PFS rates at three and five years were 76.9% and 75.1%, respectively. In the univariate analysis, OS was significantly associated with Ann Arbor stage (p = 0.03) and interim PET scan response (p = 0.01). PFS was significantly influenced by the interim PET response (p < 0.001) and treatment escalation (p = 0.01). In the multivariate analysis, bulky disease (p = 0.003) and consolidation radiotherapy (p = 0.02) were significant predictors of OS.

Patients who did not undergo interim PET scans were excluded from a subgroup analysis (153 out of 169; 90.5%). In this subgroup, the univariate analysis revealed that a positive interim PET scan was significantly associated with poorer OS (p = 0.002), while a negative scan was correlated with better PFS (p < 0.001). Treatment modality and delays also impacted PFS. In multivariate analysis, radiotherapy (p = 0.02), bulky disease (p = 0.006), and interim PET response (p = 0.007) were significantly associated with OS. PFS remained significantly affected only by the interim PET status (p = 0.005).

The prognostic value of interim PET scans has been previously demonstrated by Hutchings M et al., who identified them as independent predictors of both OS and PFS [13], findings supported by other studies [14]. Our results further support the use of interim PET scans for prognosis.

Among patients with positive interim PET scans, we compared OS and PFS between those who received escalated therapy and those who continued with the baseline regimen. No statistically significant differences were found in OS (p = 0.06) or PFS (p = 0.51) between the two groups. In the multivariate analysis, histological subtype was significantly associated with PFS (p = 0.001), but no other factors significantly affected survival. Previous research on treatment escalation following a positive PET scan has produced mixed results. Some studies support intensification to improve survival [15,16], while others, such as that by Zheng S et al. [17], have shown that continuing ABVD can still yield favorable outcomes. A 2020 European multicenter study also concluded that ABVD offers similar survival with lower toxicity compared to intensified regimens [18]. Likewise, Russell J et al.found no survival benefit from escalation [19]. Our findings are in line with these studies.

In the interim PET-negative group, over 75% of the patients received bleomycin-containing regimens (ABVD/BEACOPP), while approximately 25% received regimens without bleomycin (AVD). Continuing bleomycin in this subgroup did not significantly affect OS (p = 0.97) or PFS (p = 0.96). No factor significantly influenced OS in the univariate analysis. However, the IPS was significantly associated with PFS (p = 0.02). In the multivariate analysis, bulky disease (p = 0.01) and radiotherapy (p = 0.01) were significant predictors of PFS. OS was not significantly impacted by any factor. Similar findings were reported by Soldi LR et al., who found no survival benefit from continuing bleomycin [20]. By contrast, Al Hadidi et al. reported poorer outcomes when bleomycin was omitted due to toxicity [21].

We compared our results with those of the RATHL trial. Our cohort included 169 patients, fewer than the RATHL trial. The male-to-female ratio in our study was 1.86:1, compared to 1.2:1 in RATHL. Stage IV disease was more prevalent in our cohort (57.4% vs. 28.3%), while the rates of bulky disease were similar (32.5% vs. 32.1%). B symptoms were more common in our population (70.4% vs. 61.3%), and a higher percentage of our patients had an IPS score ≥4.

On interim PET/CT (NCAP), a higher proportion of RATHL patients achieved complete remission. However, disease progression was less frequent in our cohort (0.6% vs. 3.4%). Among PET-negative patients, 75% in our study continued ABVD, compared to 50% in RATHL. Bleomycin was discontinued in 50% of RATHL patients but only 19% in our study, due to concerns over pulmonary toxicity (e.g., respiratory symptoms, more than 10% reduction in DLCO-Hb). In addition, more patients in our study received radiotherapy than in RATHL (34.6% vs. 3.4%). In the PET-positive group, none of our patients received BEACOPP-14, compared to 51% in the RATHL trial. A larger proportion of our patients continued ABVD (42.9% vs. 2.2%), and more received radiotherapy (49% vs. 10.9%). While RATHL escalated 94.5% of patients to BEACOPP-14 or similar regimens, we escalated only 34.7%.

Despite these differences, PFS was comparable between our study and the RATHL trial. However, OS was lower in our cohort, likely due to the higher rate of advanced-stage disease. When comparing OS and PFS across the entire cohort, the PET-positive group, and the PET-negative group (with or without bleomycin), we found no statistically significant differences in survival compared to RATHL. While the RATHL trial found that the Ann Arbor stage (p = 0.001), IPS score (p = 0.029), and age (p = 0.012) significantly influenced PFS in PET-negative patients, we did not observe such associations in our study (IPS p = 0.05, stage p = 0.63, age p = 0.75).

Strengths and limitations

A key strength of our study is its focus on CHL, a common and clinically significant cancer. In routine clinical practice, many patients are not suitable for BEACOPP-escalated therapy due to factors such as age, comorbidities, or treatment-related side effects. In such cases, continuing with ABVD remains a practical and safe alternative. Our findings support this approach and demonstrate that ABVD can yield comparable survival outcomes. This highlights an important real-world consideration, showing that treatment can be safely tailored to patient needs, particularly those with limited disease or reduced tolerance, without compromising effectiveness.

However, our study also has limitations. The sample size was smaller. In particular, in the subgroups, we had a smaller number of patients than in the RATHL trial. The same is true for the PET-negative group. We de-escalated a significantly smaller number of patients to AVD. The number of events, especially deaths in our population, was also lower than in the RATHL trial. In addition, some patients were excluded due to the absence of interim PET scans. These factors may limit the generalizability and statistical strength of our conclusions. Nonetheless, considering this a single-center study, the cohort was sufficient to provide meaningful insights. Larger studies with more participants and clinical events are needed to confirm and expand upon these findings.

## Conclusions

Our real-world data from a low-to-middle-income country demonstrate excellent outcomes in the treatment of CHL using a PET-directed approach similar to the RATHL trial. A significant number of patients in our cohort presented with advanced-stage disease (stage IV), B symptoms, and high IPSs (IPS ≥ 4). Despite adherence to a PET-guided protocol, interim PET positivity was significantly linked to poorer PFS and OS. By contrast, the presence of non-bulky disease and the use of consolidation radiotherapy were associated with improved OS, although they did not significantly impact PFS. Importantly, treatment de-escalation to AVD in patients with negative interim PET scans did not compromise either OS or PFS. Among those with positive interim PET results, patients who continued ABVD due to drug tolerance concerns or near-complete response with only a single residual site achieved survival outcomes comparable to those who underwent escalation. Based on these findings, we conclude that continuing ABVD in selected interim PET-positive patients, particularly those showing good clinical response or limited residual disease, is a safe and effective approach that does not compromise survival outcomes.
